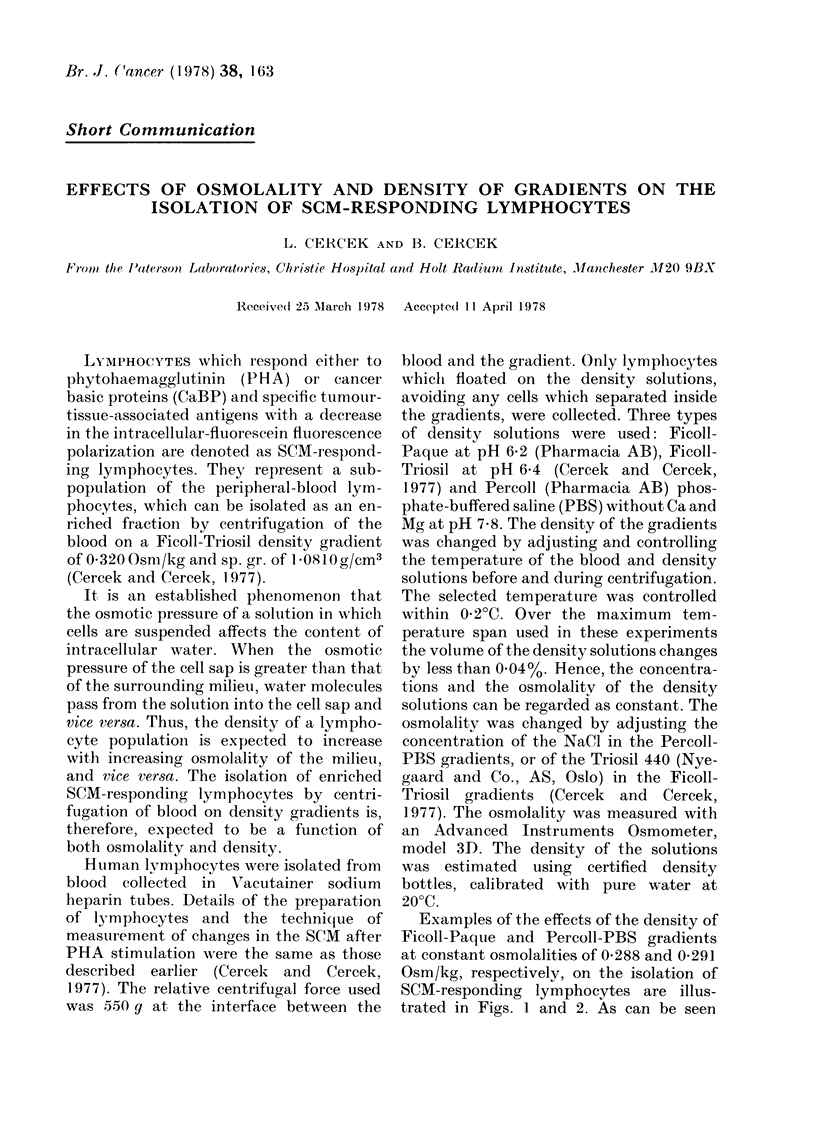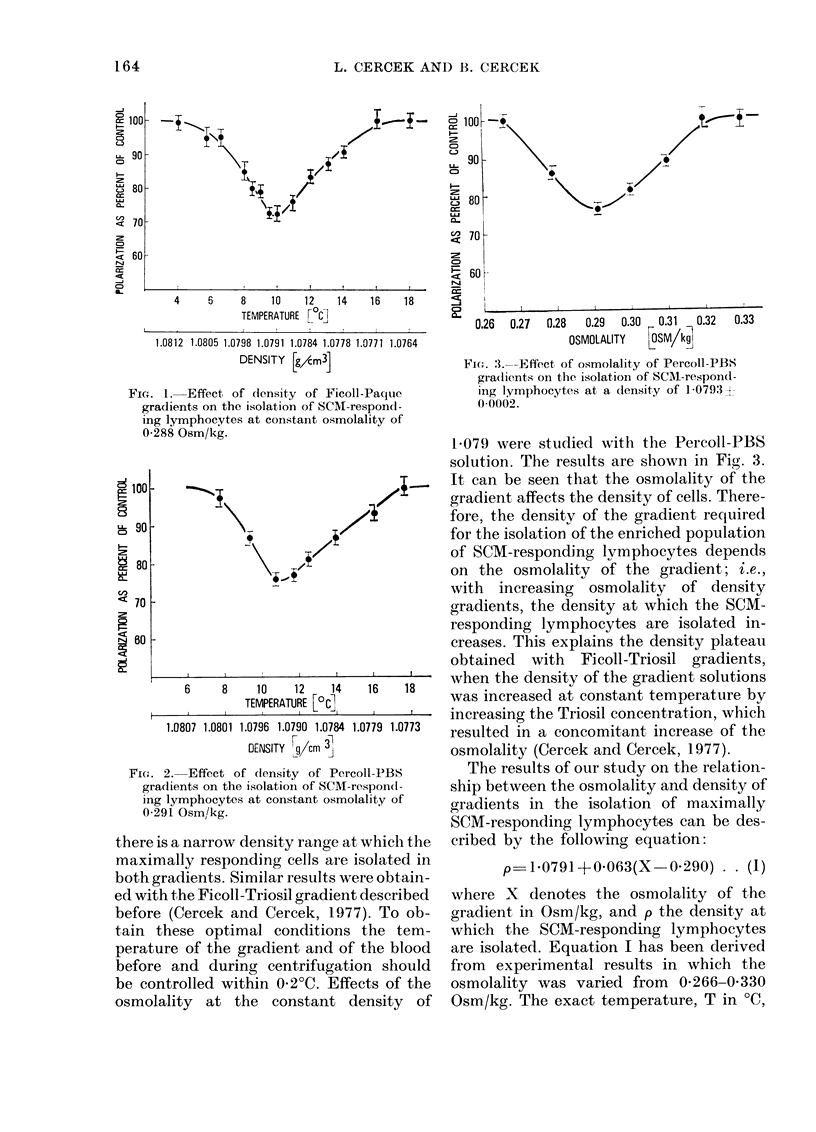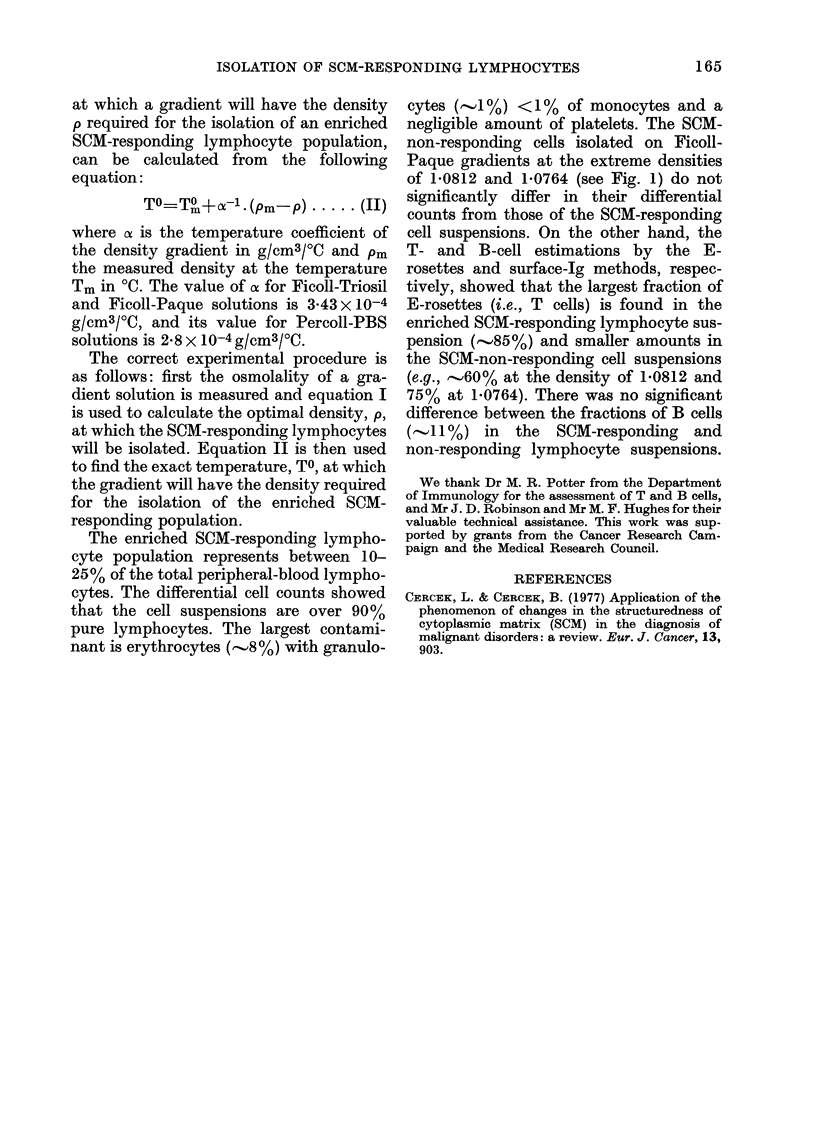# Effects of osmolality and density of gradients on the isolation of SCM-responding lymphocytes.

**DOI:** 10.1038/bjc.1978.179

**Published:** 1978-07

**Authors:** L. Cercek, B. Cercek


*Br. J. Cancer* (1978) **38**, 163

***Short Communication***

### EFFECTS OF OSMOLALITY AND DENSITY OF GRADIENTS ON THE ISOLATION OF SCM-RESPONDING LYMPHOCYTES

L. CERCEK AND B. CERCEK

*From the Paterson Laboratories, Christie Hospital and Holt Radium Institute, Manchester M20 9BX*

Received 25 March 1978 Accepted 11 April 1978

LYMPHOCYTES which respond either to phytohaemagglutinin (PHA) or cancer basic proteins (CaBP) and specific tumour-tissue-associated antigens with a decrease in the intracellular-fluorescein fluorescence polarization are denoted as SCM-responding lymphocytes. They represent a subpopulation of the peripheral-blood lymphocytes, which can be isolated as an enriched fraction by centrifugation of the blood on a Ficoll-Triosil density gradient of 0·320 Osm/kg and sp. gr. of 1·0810 g/cm$^3$ (Cercek and Cercek, 1977).

It is an established phenomenon that the osmotic pressure of a solution in which cells are suspended affects the content of intracellular water. When the osmotic pressure of the cell sap is greater than that of the surrounding milieu, water molecules pass from the solution into the cell sap and *vice versa*. Thus, the density of a lymphocyte population is expected to increase with increasing osmolality of the milieu, and *vice versa*. The isolation of enriched SCM-responding lymphocytes by centrifugation of blood on density gradients is, therefore, expected to be a function of both osmolality and density.

Human lymphocytes were isolated from blood collected in Vacutainer sodium heparin tubes. Details of the preparation of lymphocytes and the technique of measurement of changes in the SCM after PHA stimulation were the same as those described earlier (Cercek and Cercek, 1977). The relative centrifugal force used was 550 *g* at the interface between the blood and the gradient. Only lymphocytes which floated on the density solutions, avoiding any cells which separated inside the gradients, were collected. Three types of density solutions were used: Ficoll-Paque at pH 6·2 (Pharmacia AB), Ficoll-Triosil at pH 6·4 (Cercek and Cercek, 1977) and Percoll (Pharmacia AB) phosphate-buffered saline (PBS) without Ca and Mg at pH 7·8. The density of the gradients was changed by adjusting and controlling the temperature of the blood and density solutions before and during centrifugation. The selected temperature was controlled within 0·2°C. Over the maximum temperature span used in these experiments the volume of the density solutions changes by less than 0·04%. Hence, the concentrations and the osmolality of the density solutions can be regarded as constant. The osmolality was changed by adjusting the concentration of the NaCl in the Percoll-PBS gradients, or of the Triosil 440 (Nyegaard and Co., AS, Oslo) in the Ficoll-Triosil gradients (Cercek and Cercek, 1977). The osmolality was measured with an Advanced Instruments Osmometer, model 3D. The density of the solutions was estimated using certified density bottles, calibrated with pure water at 20°C.

Examples of the effects of the density of Ficoll-Paque and Percoll-PBS gradients at constant osmolalities of 0·288 and 0·291 Osm/kg, respectively, on the isolation of SCM-responding lymphocytes are illustrated in Figs. 1 and 2. As can be seen

FIG. 1.—Effect of density of Ficoll-Paque gradients on the isolation of SCM-responding lymphocytes at constant osmolality of 0·288 Osm/kg.

FIG. 2.—Effect of density of Percoll-PBS gradients on the isolation of SCM-responding lymphocytes at constant osmolality of 0·291 Osm/kg.

there is a narrow density range at which the maximally responding cells are isolated in both gradients. Similar results were obtained with the Ficoll-Triosil gradient described before (Cercek and Cercek, 1977). To obtain these optimal conditions the temperature of the gradient and of the blood before and during centrifugation should be controlled within 0·2°C. Effects of the osmolality at the constant density of

FIG. 3.—Effect of osmolality of Percoll-PBS gradients on the isolation of SCM-responding lymphocytes at a density of 1·0793 ± 0·0002.

1·079 were studied with the Percoll-PBS solution. The results are shown in Fig. 3. It can be seen that the osmolality of the gradient affects the density of cells. Therefore, the density of the gradient required for the isolation of the enriched population of SCM-responding lymphocytes depends on the osmolality of the gradient; *i.e.*, with increasing osmolality of density gradients, the density at which the SCM-responding lymphocytes are isolated increases. This explains the density plateau obtained with Ficoll-Triosil gradients, when the density of the gradient solutions was increased at constant temperature by increasing the Triosil concentration, which resulted in a concomitant increase of the osmolality (Cercek and Cercek, 1977).

The results of our study on the relationship between the osmolality and density of gradients in the isolation of maximally SCM-responding lymphocytes can be described by the following equation:

$$\rho = 1{\cdot}0791 + 0{\cdot}063(X - 0{\cdot}290) \quad . \quad . \quad (I)$$

where X denotes the osmolality of the gradient in Osm/kg, and $\rho$ the density at which the SCM-responding lymphocytes are isolated. Equation I has been derived from experimental results in which the osmolality was varied from 0·266–0·330 Osm/kg. The exact temperature, T in °C, at which a gradient will have the density $\rho$ required for the isolation of an enriched SCM-responding lymphocyte population, can be calculated from the following equation:

$$T^0 = T^0_m + \alpha^{-1} \cdot (\rho_m - \rho) \quad \ldots\ldots \text{(II)}$$

where $\alpha$ is the temperature coefficient of the density gradient in g/cm$^3$/°C and $\rho_m$ the measured density at the temperature $T_m$ in °C. The value of $\alpha$ for Ficoll-Triosil and Ficoll-Paque solutions is $3{\cdot}43 \times 10^{-4}$ g/cm$^3$/°C, and its value for Percoll-PBS solutions is $2{\cdot}8 \times 10^{-4}$ g/cm$^3$/°C.

The correct experimental procedure is as follows: first the osmolality of a gradient solution is measured and equation I is used to calculate the optimal density, $\rho$, at which the SCM-responding lymphocytes will be isolated. Equation II is then used to find the exact temperature, $T^0$, at which the gradient will have the density required for the isolation of the enriched SCM-responding population.

The enriched SCM-responding lymphocyte population represents between 10–25% of the total peripheral-blood lymphocytes. The differential cell counts showed that the cell suspensions are over 90% pure lymphocytes. The largest contaminant is erythrocytes (~8%) with granulocytes (~1%) <1% of monocytes and a negligible amount of platelets. The SCM-non-responding cells isolated on Ficoll-Paque gradients at the extreme densities of 1·0812 and 1·0764 (see Fig. 1) do not significantly differ in their differential counts from those of the SCM-responding cell suspensions. On the other hand, the T- and B-cell estimations by the E-rosettes and surface-Ig methods, respectively, showed that the largest fraction of E-rosettes (*i.e.*, T cells) is found in the enriched SCM-responding lymphocyte suspension (~85%) and smaller amounts in the SCM-non-responding cell suspensions (*e.g.*, ~60% at the density of 1·0812 and 75% at 1·0764). There was no significant difference between the fractions of B cells (~11%) in the SCM-responding and non-responding lymphocyte suspensions.

We thank Dr M. R. Potter from the Department of Immunology for the assessment of T and B cells, and Mr J. D. Robinson and Mr M. F. Hughes for their valuable technical assistance. This work was supported by grants from the Cancer Research Campaign and the Medical Research Council.

### REFERENCES

Cercek, L. & Cercek, B. (1977) Application of the phenomenon of changes in the structuredness of cytoplasmic matrix (SCM) in the diagnosis of malignant disorders: a review. *Eur. J. Cancer*, **13**, 903.